# Pan-Resistome Characterization of Uropathogenic *Escherichia coli* and *Klebsiella pneumoniae* Strains Circulating in Uganda and Kenya, Isolated from 2017–2018

**DOI:** 10.3390/antibiotics10121547

**Published:** 2021-12-17

**Authors:** Arun Gonzales Decano, Kerry Pettigrew, Wilber Sabiiti, Derek J. Sloan, Stella Neema, Joel Bazira, John Kiiru, Hellen Onyango, Benon Asiimwe, Matthew T. G. Holden

**Affiliations:** 1 School of Medicine, University of St Andrews, St Andrews KY16 8BQ, UK; global.challenges@essb.eur.nl (K.P.); ws31@st-andrews.ac.uk (W.S.); djs26@st-andrews.ac.uk (D.J.S.); mtgh@st-andrews.ac.uk (M.T.G.H.); 2 Department of Sociology and Anthropology, Makerere University, Kampala P.O. Box 7062, Uganda; sheisim@yahoo.com; 3 Faculty of Medicine, Mbarara University of Science and Technology, Mbarara P.O. Box 410, Uganda; jbazira@gmail.com; 4 Centre of Microbiology Research, Kenya Medical Research Institute, Off Raila Odinga Way, Nairobi P.O. Box 54840 00200, Kenya; kyirow@gmail.com; 5 Department of Medical Microbiology, Jomo Kenyatta University of Agriculture and Technology, Nairobi P.O. Box 62 000, Kenya; hao1@st-andrews.ac.uk; 6 Department of Medical Microbiology, Makerere University College of Health Sciences, Kampala P.O. Box 7062, Uganda; benon.asiimwe@gmail.com

**Keywords:** antimicrobial resistance, pangenome, lmic, public health

## Abstract

Urinary tract infection (UTI) develops after a pathogen adheres to the inner lining of the urinary tract. Cases of UTIs are predominantly caused by several Gram-negative bacteria and account for high morbidity in the clinical and community settings. Of greater concern are the strains carrying antimicrobial resistance (AMR)-conferring genes. The gravity of a UTI is also determined by a spectrum of other virulence factors. This study represents a pilot project to investigate the burden of AMR among uropathogens in East Africa. We examined bacterial samples isolated in 2017–2018 from in- and out-patients in Kenya (KY) and Uganda (UG) that presented with clinical symptoms of UTI. We reconstructed the evolutionary history of the strains, investigated their population structure, and performed comparative analysis their pangenome contents. We found 55 *Escherichia coli* and 19 *Klebsiella pneumoniae* strains confirmed uropathogenic following screening for the prevalence of UTI virulence genes including *fimH*, *iutA*, *feoA*/*B*/*C*, *mrkD,* and *foc*. We identified 18 different sequence types in *E. coli* population while all *K. pneumoniae* strains belong to ST11. The most prevalent *E. coli* sequence types were ST131 (26%), ST335/1193 (10%), and ST10 (6%). Diverse plasmid types were observed in both collections such as Incompatibility (IncF/IncH/IncQ1/IncX4) and Col groups. Pangenome analysis of each set revealed a total of 2862 and 3464 genes comprised the core genome of *E. coli* and *K. pneumoniae* population, respectively. Among these are acquired AMR determinants including fluoroquinolone resistance-conferring genes *aac(3)-Ib-cr* and other significant genes: *aad*, *tet*, *sul1*, *sul2*, and *cat,* which are associated with aminoglycoside, tetracycline, sulfonamide, and chloramphenicol resistance, respectively. Accessory genomes of both species collections were detected several β-lactamase genes, *bla*_CTX-M_, *bla*_TEM_ and *bla*_OXA,_ or *bla*_NDM_. Overall, 93% are multi-drug resistant in the *E. coli* collection while 100% of the *K. pneumoniae* strains contained genes that are associated with resistance to three or more antibiotic classes. Our findings illustrate the abundant acquired resistome and virulome repertoire in uropathogenic *E. coli* and *K. pneumoniae,* which are mainly disseminated via clonal and horizontal transfer, circulating in the East African region. We further demonstrate here that routine genomic surveillance is necessary for high-resolution bacterial epidemiology of these important AMR pathogens.

## 1. Introduction

Antimicrobial resistance (AMR) has raised alarms as a global health threat. AMR is often fueled by misuse and abuse of antibiotics including self-medication [1,2] and unrestricted access to antimicrobial drugs [3,4,5], and is further accelerated by industrialization, poor waste disposal, and poor hygiene levels. AMR pathogens are frequently detected in food, clinical, and environmental settings in East Africa. Despite facing broad challenges, significant efforts have recently been put in place to curb AMR in East African countries. For instance, Kenya (KY) has adapted the National Action Plan that incorporates One Health measures to prevent AMR and is highly supported by multiple governmental policies (NAPCAR 2017) [6]. Similarly, an extensive evaluation of the AMR situation in Uganda (UG) was assessed by the Uganda National Academy of Sciences (UNAS) supported by the Global Antibiotic Resistance Partnership (GARP)-Uganda (UNAS 2015) [7]. High prevalence of multi-drug resistant bacteria particularly extended-spectrum beta-lactamase (ESBL)-producing strains is significantly recorded in both countries.

Urinary tract infection (UTI) develops after a pathogen’s adherence to the inner lining of the urinary tract. UTIs occur among patients of all age groups and account for high morbidity in the clinical and community settings [8]. Following binding within the urinary tract, uropathogens either cause asymptomatic or commensal connection or severe disease. About 1% of the population have asymptomatic bacteriuria (ABU), wherein a pathogen (≥10^5^ cfu mL^−1^) inhabits the tract without eliciting mucosal host response [9,10]. Infections in the lower urinary tract region (e.g., cystitis) are recognized by symptoms such as dysuria. Successful virulent strains can induce pyelonephritis where rapid immune response is mobilized via cytokine secretion and influx of immune cells. UTIs are either uncomplicated or complicated. Uncomplicated UTI cases are usually observed in patients who are otherwise healthy, while complicated UTIs are diagnosed in compromised patients (e.g., if they have anatomical or functional anomalies in their urinary tract or are under long-term catheterization) [11] Treatment of these complicated UTI cases is often confounded by AMR uropathogens usually caused by Gram-negative bacteria [12]. Uncomplicated UTIs are frequently caused by uropathogenic *Escherichia coli* (*E. coli* (UPEC)) while complicated cases might be caused by several pathogens such as *Proteus mirabilis, Providencia stuartii, Morganella morganii, Klebsiella pneumoniae (K. pneumoniae)*, and *Pseudomonas aeruginosa* [8]. Recurrent UTI cases are also common, particularly when urinary tract anomalies linger, or treatment failed to kill resistant bacteria [13], leading to more severe type of infections. Due to the lack of active investigation of UTI cases in East Africa, particularly in the community, access to accurate data can be challenging.

An increasing number of studies have employed whole genome sequencing (WGS) and analyses for disease surveillance in both hospital and community settings [14,15,16]. The high-resolution genotyping that WGS provides allows one to investigate and describe the population structure and evolutionary history of the isolates, as well as tracing their spread. Outbreaks have been robustly detected and described using high-throughput methodologies designed for bacterial pathogens [17,18,19,20]. Comprehensive AMR gene databases and prediction tools are also available that help assess AMR gene content in whole genomes with high accuracy [21].

Here, we used WGS to investigate the prevalence of acquired AMR-conferring genes in *E. coli* and *K. pneumoniae* isolated from urine samples taken from patients in rural areas of KY and UG that presented UTI-like symptoms. Our analysis of AMR determinants was limited to those associated with the pan genome and mutation in core genes responsible for antibiotic resistance were not investigated. We further explored their phylogenetic relationships of the isolates collected with other currently circulating African and global strains. This study represents a pilot project of the HATUA consortium. HATUA stands for Holistic Approach to Unravel Antibiotic Resistance in East Africa and the team is comprised of researchers from different disciplines that aim to tackle the main drivers of AMR among uropathogens in East Africa.

## 2. Methods

### 2.1. Study Design and Patient Recruitment

A total of N = 150 bacterial isolates were obtained from patients in KY (*n* = 91) and UG (*n* = 59) presenting UTI-like symptoms, as part of a larger study. Ethical Review Board of University of St Andrews ethical approval, Approval code MD14548 and KY (KEMRI/SERU/P00112/3865) approved verbal consent taken from all the patients. Important patient data such as name, age, gender, location was recorded, and unique identification number were assigned to each patient.

### 2.2. Library Preparation and Whole Genome Sequencing

Bacterial genomic DNA for the isolates were extracted using the QIAxtractor (Qiagen, Valencia, CA, USA) according to the manufacturer’s instructions. Library preparation was conducted according to the Illumina protocol and sequenced (96-plex) on an Illumina MiSeq platform (Illumina, San Diego, CA, USA) using 250 bp paired-end reads.

### 2.3. Read Library Quality Control, Mapping and De Novo Genome Assembly

Illumina MiSeq read libraries were rid of sequencing adapters and ambiguous bases using Fastp [22]. Sets that passed the quality filtering were de novo assembled using Unicycler v4.6 [23] pipeline in normal mode to merge contigs.

The read libraries were mapped to reference sequences using SMALT v7.6 (http://www.sanger.ac.uk/resources/software/SMALT/ (accessed on 18 December 2019) [24] and the resulting SAM files were converted to BAM format, sorted and PCR duplicates removed using SAMtools v1.19 [25]. Strain TOP52_1721_U1 [26] was used the reference genome for the *K. pneumoniae* samples while the strain EC958 [27] was employed as the reference sequence for the *E. coli* population.

### 2.4. Species Identification, Sero- and Sequence Typing, Genome Annotation and Screening for UTI Virulence/AMR Genes

Prediction of bacterial species was carried out by uploading the assemblies on PathogenWatch website (https://pathogen.watch (accessed on 31 October 2019)) [28], which runs Speciator (https://gitlab.com/cgps/mash-speciator (accessed on 31 October 2019)) for its species assignment. Speciator employs Mash [29] to identify the most identical strain (≥90% identity) in a reference collection of complete genomes found in the NCBI RefSeq database (https://www.ncbi.nlm.nih.gov/refseq/ (accessed on 31 October 2019)) [30]. The strains are then grouped according to their species designation and were screened for UTI pathogen determinants. Multi-locus sequence typing was performed by running SRST2 v.0.2.0 [31] based on the Achtman scheme [32] for *E. coli* and Pasteur [33] for *K. pneumoniae* isolates. Antigenic (O polysaccharide and H flagellin) profiles of *E. coli* samples were identified by employing Serotypefinder v.2.0 (https://cge.cbs.dtu.dk/services/SerotypeFinder/ (accessed on 13 December 2019)) [34] at 85% ID threshold and 60% minimum length.

Genome composition of the draft assemblies was assessed using Prokka v.1.10 [35] Acquired AMR genes were identified by aligning the genome sequences to the 2158 gene homolog subset of the Comprehensive Antibiotic Resistance Database (CARD) v. 3.0.8 (https://card.mcmaster.ca/ (accessed on 08 November 2019)) [36] and BacWGSTdb 2.0 [37] Clustering based on the distribution of AMR genes among isolates was drawn using Phandango v.1.3.0 [38]. Plasmid and replicon typing was carried out by comparing the genomes against the PlasmidFinder database v. 2.1.1 [39] at 99% identity threshold.

### 2.5. Bacterial Sample Collection and Antimicrobial Susceptibility Testing

To determine concordance between the AMR gene content and sample phenotype, antibiotic susceptibility testing and phenotypic detections of ESBL were performed by disc diffusion methods on a subset of *n* = 16 isolates from KY. The tests were carried out according to CLSI (2016) guidelines [40]. Isolates were examined for the insusceptibility to 9 different classes of antibiotics including Penicillin (ampicillin (AMP)), Penicillin + β-lactamase inhibitors (ampicillin-clavulanic acid (AMC)), Chloramphenicol (Chloramphenicol (CHL)), Sulfonamide (Trimethoprim-sulfamethoxazole (SXT)) and Quinolones (nalidixic acid (NA)), and Fluoroquinolone (Ciprofloxacin (CIP)). Resistance to ESBL Cephalosphorins was also assessed by testing the strains with Ceftriaxone (CRO), Ceftazidime (CAZ), cefotaxime (CTX), and Cefepime (FEP) (Supplementary Table S1).

### 2.6. Phylogenetic Reconstruction

Phylogenetic relationships and sequence variations between the samples were determined by constructing phylogenetic trees based on their chromosomal single-nucleotide polymorphism (SNP)s. Mobile genetic elements (MGEs) were further excluded using an internal script. Non-recombinant SNPs were determined using ClonalframeML v. 1.12 [41] and were used to create a maximum-likelihood midpoint-rooted phylogeny using RAxML v8.0.19 [42] using a General Time Reversible + gamma (GTR + G) substitution model with 100 bootstraps. Phylogenies were visualized using iToL (https://itol.embl.de/ (accessed on 20 January 2020)) [43] and FigTree v1.4.3 (http://tree.bio.ed.ac.uk/software/figtree/ (accessed on 20 January 2020)) [44].

### 2.7. Pangenome Analyses

The resulting annotation files from Prokka v.1.10 [35] were used as the basis for generating a pangenome for each species set. This step was completed by running Roary v3.11.2 [45] with a 100% BLAST v2.6.0 identity threshold using the MAFFT v7.3 setting [46]. Pangenome outputs were also used to assess the accessory genome composition of each bacterial population and as basis for reconstructing core genome phylogenies.

## 3. Results

### 3.1. Patient and Bacterial Strain Profiles

From the total of N = 150 strains, we collected from urine samples of patients, *n* = 81 were identified as *E. coli* and *n* = 19 were *K. pneumoniae.* The respondents were either to be admitted or visiting rural hospitals in KY and from clinics in the countryside of UG.

### 3.2. Genomic and Pangenomic Characterization Confirmed the Virulence Factors Present in Uropathogenic E. coli and K. pneumoniae

A subset (*n* = 55) from the total *n* = 81 *E. coli* and all *n* = 19 *K. pneumoniae* were confirmed uropathogenic following a thorough characterization of their pangenome contents. One thousand one hundred forty-four (1144) and 3464 core genes were found across the strains in *E. coli* and *K. pneumoniae* populations, respectively. These include known UTI virulence markers that are responsible for urinary tract (mucosal) surface binding (type 1 fimbrial adhesin-coding *fimH*) and colonization (*mrkD*; *K. pneumoniae* only), iron (Fe(2+)) transport (*feoA/B/C*), enterobactin synthase production (*entB*), formate transport (*focA*), cell division (*zapA*), succinate-acetate/proton symport (*satP*), anaerobic sulfatase-maturation (*chuR*; found in 100% and 95% of *E. coli* and *K. pneumoniae*, respectively). Other important virulence genes were also found, albeit not conserved among all the isolates: *iutA* (ferric aerobactin receptor: 44.6% in *E. coli*, 10% in *K. pneumoniae*), *papA* (fimbrial major pilin protein: *E. coli* only (41%)), *papD* (import of P pilus subunits into the periplasm: 44.6% in *E. coli*, 10% in *K. pneumoniae*), *hlyE* (hemolysin E: 80.4% in *E. coli*, 10% in *K. pneumoniae*), *fyuA* (pesticin receptor: 73.2% in *E. coli*, 15% in *K. pneumoniae*), *kpsT* (polysialic acid transport ATP-binding protein: *E. coli* only (26.8%)) and *pic* (serine protease pic autotransporter: *E. coli* only (5.4%); Table 1).

### 3.3. Prevalence of AMR Genes in E. coli and K. pneumoniae Uropathogens from KY and UG

All *n* = 55 *E. coli* and *n* = 19 *K. pneumoniae* isolates harbored type 1 fimbrin. Among the UPEC, *fimH30* was the most common allele, followed by *fimH41*; *n* = 4/55 samples had type *fimH22* and *n* = 2/55 singleton were found with *fimH22*.

We further detected multiple acquired AMR-conferring elements in the genomes of the two species collections. Alignment of the sequences against CARD v.3.0.7 with 98–100% identity revealed that the *n* = 55 *E. coli* (*n* = 31 from KY, *n* = 24 from UG) were detected with the ciprofloxacin-conferring gene, *marA*. Majority (*n* = 47/55) were also aminoglycoside resistant and harbors either *aadA* or *aac(6′)-Ib/(3′)-Ib* alleles or both. Only *n* = 11/55 were not detected with resistant genes for ESBL cephalosphorins. Of the *n* = 44/55 that produce ESBLs, *n* = 10/44 had *bla*_CTX-M_ (allele type 15 or 88), *n* = 24/44 had *bla*_TEM_ (type 30/2/220) and *n* = 9/44 had *bla*_OXA-1/140_ and *n* = 2/44 (both from UG) had all 3 ESBL genes. Sulfonamide resistance genes were widely observed, *n* = 39 had either *sul1* only, *sul2* only or *sul3* only, or both *sul1* and *sul2*. Tetracycline resistance gene, *tet(A)* was present in *n* = 34 of 55. Twenty-four (N = 24/55) contained macrolide resistance-conferring *mphA*, *n* = 9/55 (*n* = 6 from KY, *n* = 3 from UG) harbored *catB3* and were chloramphenicol resistant. Ninety-five percent (95%, *n* = 52/55) had at least one gene that codes for efflux pump proteins with *n* = 4/55 having *yojI-pmrF-emrR-bacA*-*acrS/B/E-msbA-evgA-kdpE-mdtP-eptA* cassette and *n* = 1/55 containing a mixture of *yojI, pmrF*, *emrR*, *bacA*, *acrS/B/E*, *msbA*, *evgA*, *kdpE*, *mdtP*, *eptA*, *emtK*, *cpxA* (Table 2 and Table 3). Two KY isolates (71 and 72) were found to have the fluoroquinolone resistance-conferring gene *aac(3′)-Ib-cr* while its variant *aac(6′)-Ib-cr* was present in UG isolates BN19, BN38 and BN44 (Supplementary Figure S1a).

All *n* = 19 *K. pneumoniae* isolates were resistant to aminoglycosides and had either *aac(6′)*-30/*aac(6′)*-Ib’/7/10-*aadA9* (*n* = 10/19) or *aac(6′)*-Ib7*-aadA9* (*n* = 1/19) combination. N = 18/19 are potentially sulfonamide insusceptible and contained either *sul*1 only (*n* = 2/19), *sul*2 only (*n* = 15/19) or both (*n* = 1). The β-lactamase *bla*_LEN_ gene is present in all but the BN7 strain, with *bla*_LEN-4/6_ (*n* = 15 from KY) or *bla*_LEN-3/4/5/6_ alleles. BN7 was also the only susceptible isolate against ESBL cephalosphorins. The rest are ESBL producers: *n* = 15/19 had *bla*_SHV-28_, *bla*_CTX-M-15_, *bla*_OXA-1/140_ and *bla*_NDM-1_, *n* = 1/19 were observed with the *bla*_SHV-28-CTX-M-15-OXA-1/140_ combination and *n* = 1/19 had *bla*_SHV-28_, *bla*_CTX-M-15/88_ only. All *n* = 3 strains from UG had *tet(B)* and *dfrA* (17 or 27 allele type); these strains also contained efflux pump-expressing genes: *yojI*, *pmrF*, *emrR*, *bacA*, *acrB*, *msbA*, *evgA*, *kdpE*, *mdtP*, *eptA*, *emtK*, and *cpxA*. N = 2/19 (BN14 and BN16 from UG) had ciprofloxacin-resistance gene, *marA*. Only the strain 90 from KY was not resistant to phenicols, while the rest were detected with the *cat* gene, specifically, *catB3* (Table 3; Supplementary Figure S2b). Overall, 80% of our *E. coli* uropathogens had ESBL genes (*n* = 15 strains from UG and *n* = 29 from KY) and 93% of these UPEC are MDR, while all the *K. pneumoniae* isolates are MDR and only *n* = 1 out of the total *n* = 19 (95%) are ESBL.

### 3.4. Population Structure of KY and UG Uropathogens

**Figure 1:**
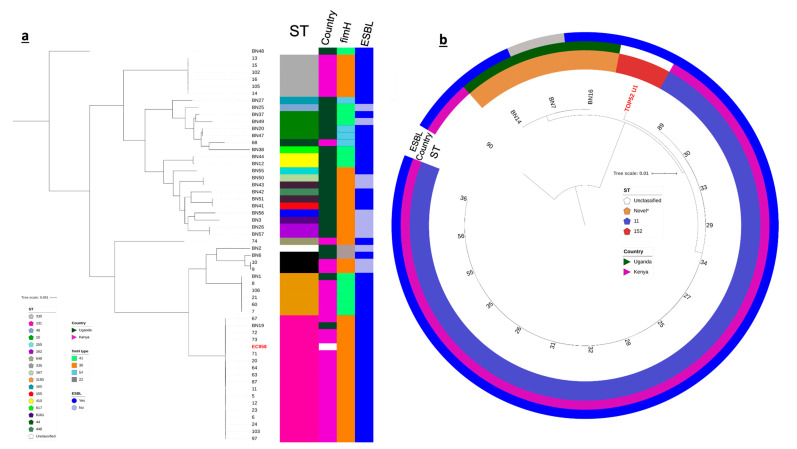
Maximum likelihood phylogenies of core genomes of *E. coli* (**a**) and *K. pneumoniae* (**b**) uropathogens isolated from KY and UG; reference genomes are in red and font. The mid-point rooted phylograms was constructed using 2862 and 3464 core genes from *E. coli* and *K. pneumoniae* populations, respectively and visualized with iTol. The colored strips adjacent to the *E. coli* phylogeny represent (from left to right) the sequence type (ST), country of origin, type of *fimH* allele and the ESBL status of each strain. The colored rings around the *K. pneumoniae* phylogenetic tree indicate the ST, country of origin and the ESBL status of each isolate. “Novel*” means the sequence type of the sample/s did not match those in the database and may be novel. The scale bar indicates substitutions per site.

The UPEC collection was polyclonal. Eighteen (18) different sequence types were identified in the UPEC population (Achtman scheme). The most prevalent MLST sequence types were ST131 (*n* = 17/55, 31%), ST335 and ST1193 (*n* = 6/55, 11%) and ST10 (*n* = 4/55, 7%). These sequence types were usually associated with UTI cases (Nicolas-Chanoine et a. 2014; Afset et al. 2008; Yamaji et al. 2018); the globally disseminated ESBL-ST131 stood out to be the most dominant ST. Other clones were also observed: *n* = 3 ST73, *n* = 2 each from ST155, ST410, ST6161 and ST162, and singletons from ST44, ST48, ST165, ST167, ST212, ST448, ST617, ST648 and ST2163; *n* = 2 strains from UG (BN2 and BN48) were unclassified (Figure 1a, Table 2). *E. coli* isolates from UG belong to 15 STs and were thus more diverse compared to those collected from KY, which belong to only 6 STs (Figure 1a, Table 2). This difference in diversity is consistent with the number of serotypes found in UG relative to those from KY: Ugandan strains belong to 20 different O:H antigen combinations while the KYn ones were found to have 9 O:H types.

Fifteen out of nineteen (*n* = 15/19) *K. pneumoniae* isolates from KY belong to ST11 (Pasteur scheme); *n* = 3/19 UG had no defined sequence types (BN14: 0b8e, BN16; 67b2, BN7: 6b6f) and formed their own clade (Table 3; Figure 1b).

We compared our *E. coli* samples from the three most prevalent clones, ST131, ST335 and ST10, and our *K. pneumoniae* strains with previously published genomes listed in BacWGSTdb 2.0. Based on the metadata of the reference genomes, these strains were of different geographical origins (country/state) and were mostly isolated from human hosts and have caused disease (Supplementary Table S2). Computing for the pairwise SNP distances showed that strain CP023853 is the most closely related genome with our KY isolates with distances ranging from 910–1489; CP023853 was also sampled from a UTI patient in Sweden in 2009 (Supplementary Table S2; Supplementary Figure S2a). Our ST335 collection is solely composed of KY isolates, and all appeared to be genetically distant to the selected sequences in the database with a minimum of 4700 SNP differences between the two groups (Supplementary Figure S2a). In contrast, our *E. coli* ST10 strains were all from UG. The closest reference isolate was LSBS01 (isolated from a fecal sample; Supplementary Table S2), which was 2009 and 2070 SNPs apart from BN20 and BN70, respectively (Supplementary Figure S2a).

Our *K. pneumoniae* collection, which was dominated by ST11 showed ~3500 SNP differences from strain 27 from KY while those that had no defined ST (e.g., BN14 and BN 16 from UG) appeared to be most closely related (minimum SNP distances of 3519 and 3255) to the human isolate references LXMM01 and VUBS01, respectively (Supplementary Table S2; Supplementary Figure S2b).

### 3.5. Plasmid Characterization

Genome assemblies of the KY and UG uropathogens were screened for the presence and type of plasmids using PlasmidFinder v.2.1.1. N = 47/55 in the *E. coli* collection were found with at least one plasmid. IncFIA was consistently found in *n* = 10 had both IncFIA and IncFII, *n* = 9 contained IncFIA, IncFII, Col156 types, *n* = 1 was detected with IncFIA, IncFII and IncY only or IncI only and IncFII-IncFIA-IncX4 plasmid combinations.

All the samples from the K. pneumoniae collection were found with at least one plasmid type. IncFII-IncFIB-IncR is the most common combination and is found among *n* = 15 isolates, while *n* = 2/19 was found with IncR, IncFII, IncFIA, Col, and IncX4. Notably, the strain 90 from KY had the *bla*_CTX-M-1_ gene-carrying plasmid IncN and BN7 from UG had the *bla*_NDM_-associated IncR.

## 4. Discussion

We assessed the prevalence of acquired AMR characteristics among uropathogenic *E. coli* and *K. pneumoniae* circulating in East African region using WGS. We recruited out-patients that presented UTI-like symptoms from rural areas in KY and UG, which represents a limitation of our sample collection. The lack of point-of-care diagnostic tool such as the use of dipstick test also contributed to some difficulties in our screening. This is evidenced by a high level of contaminants that comprised of strains that do not contain UTI determinants. Nevertheless, our in silico predictions using whole genome analysis revealed alarming rates of ESBL-producing and MDR strains in both our UPEC and *K. pneumoniae* collections, which reiterates the great necessity for effective interventions to curb their spread.

Our results firmly indicate a high diversity among *E. coli* uropathogens, which was more evident in samples taken from UG rather than KY. Strains that belong to the same clonal group had <200 core SNPs from each other. This rich genetic diversity is consistent with those observed in other isolates collected from rural or semi-rural communities of low/middle-income countries [47,48,49]. The widely disseminated UTI-causing clones ST131, ST335 and ST10 were common among our *E. coli* strains and dominated our Kenyan collection. This is unsurprising as these STs are reported to be circulating globally [50,51,52]. What is remarkable is the detection of emerging clones such as ST1193 and ST617 that were unusually associated with UTI [53,54] albeit observed in hospital settings. UPEC strains from UG are even more alarming as they represent higher number of unusual or novel UTI clones (i.e., ST155, ST448, and ST162) with potentially higher virulence levels [55,56,57] compared to those globally-known STs.

Several *Klebsiella* species were known to have broad-spectrum resistance to common antibiotics [58]. *K. pneumoniae* strains particularly those belonging to the hypervirulent ST11 have been extensively reported to cause severe infections [59,60] and have led to dire disease outcomes in intensive care units [61]. This *K. pneumoniae* clone has an alarming antibiotic resistance profile [62] making it difficult to treat. The dominance of ST11 strains in our samples that were mainly collected from outpatients suggests the strong presence of this clinically important bacterial pathogen in the community and pose an apparent threat to public health.

*Bla*_CTX-M_ genes were present in 40% of our UPEC collection and in all but one *K. pneumoniae* strain (95%). Notably, the *bla*_CTX-M-15_ gene that confers resistance to last-resort antibiotics was found in high levels in both countries. This gene was detected with other ESBL determinants, *bla*_TEM_ and *bla*_OXA-1_ in *E. coli* and with *bla*_NDM_ in *K. pneumoniae,* concordant with those in uropathogens found from the Middle East [63] and Asia [64], among others. Consistent with previous findings in other African regions, *tet* genes in this study were also detected alongside ESBL genes *bla*_CTX-M-15_, *bla*_OXA-1_ and *bla*_TEM_ in *n* = 30/55 *E. coli* and with *bla*_LEN-3/4/5/6_ among *n* = 3/19 *K. pneumoniae* [65,66] which stipulates their co-selection and co-transmission in KY and UG. The presence of these genes in the identified plasmid-associated contigs suggest that the mode of transfer may have been plasmid-mediated.

## 5. Conclusions

We underline in this pilot study the high frequency of AMR determinants associated with resistance to common antibiotic classes among *E. coli* and *Klebsiella pneumoniae* in East Africa, with specific focus on MDR and ESBL-producing strains from KY and UG. We further demonstrate that routine genomic surveillance is necessary for high-resolution investigation of bacterial epidemiology especially in less represented regions. Our findings have significant implications on improving interventions that aim to address the strong presence of AMR pathogens that cause UTI (particularly in low/middle-income countries).

## Figures and Tables

**Table 1:** UTI virulence marker genes present in the pangenome of N = 55 *E. coli* and N = 19 *K. pneumoniae* isolates (including the reference genomes for each species collection). Proportion of the samples containing the gene are shown in count of strains with gene over the total strains and % values.

Gene	Protein Product	Strain Count and % in *E. coli* Collection	Strain Count and % in *K. pneumoniae* Collection
*fim* *H*	Type 1 fimbrin D-mannose specific adhesin	56/56 (100)	20/20 (100)
*feo* *A/B/C*	Fe(2+) transport protein A/B/C	56/56 (100)	20/20 (100)
*ent* *B*	Enterobactin synthase component B	56/56 (100)	20/20 (100)
*foc* *A*	Formate transporter	56/56 (100)	20/20 (100)
*zap* *A*	Cell division protein	56/56 (100)	20/20 (100)
*sat* *P*	Succinate-acetate/proton symporter	56/56 (100)	20/20 (100)
*chu* *R*	Anaerobic sulfatase-maturating enzyme	56/56 (100)	19/20 (95)
*mrk* *D*	Type 3 fimbrial adhesin	0	20/20 (100)
*hly* *E*	Hemolysin E	45/56 (80.4)	2/20 (10)
*fyu* *A*	Pesticin receptor	41/56 (73.2)	3/20 (15)
*iut* *A*	Ferric aerobactin receptor	25/56 (44.6)	2/20 (10)
*pap* *D*	Import of P pilus subunits into the periplasm	26/56 (46.4)	2/20 (10)
*pap* *A*	Fimbrial major pilin protein	23/56 (41.1)	0
*kps* *T*	Polysialic acid transport ATP-binding protein	15/56 (26.8)	0
*pic*	Serine protease pic autotransporter	3/56 (5.4)	0

**Table 2:** Genomic characteristics of uropathogenic *E. coli* strains isolated in this study for acquired AMR-associated genes and plasmid replicon types. Asterisk (*) next to the O antigen type means undefined; NF means not found; ‘Yes’ means the strain is either ESBL-producing or MDR; and ‘No’ means the sample is either non-ESBL or non-MDR.

Sample Name	Serotype	Sequenc Type Pasteur	AMR Phenotype	*fimH*	Aminoglycosides	Macrolide	Ciplrofloxacin	β-Lactamase Inhibitors/ESBL Cephalosporins	Phenicols	Fluoroquinolones	Quinolone	Sulfonamide	Tetracycline	Folate Pathway Inhibitors	Antibiotic Effulx/Regulation	ESBL	MDR	Plasmid Replicon
5	O25:H4	131	AMP, CTX, CHL, SXT, CIP, NA	H30	*aac*(3)-Ib, *aadA5*	*mphA*	*marA*	*bla*_CTX__-M-27/99_	NF	NF	NF	*sul1, sul2*	*tet(A)*	*dfrA17*	*gadW*	Yes	Yes	IncFIA, IncFII
6	O25:H4	131	AMP, CTX, CRO, CHL, SXT, CIP, NA	H30	*aac*(3)-Ib, *aadA5*	*mphA*	*marA*	*bla*_CTX__-M-27/99_	NF	NF	NF	*sul1, sul2*	*tet(A)*	*dfrA17*	*gadW*	Yes	Yes	IncFIA, IncFII
7	O*:H5	1193	CRO, CHL, SXT, CIP, NA	H41	*aac*(3)-Ib, *aadA17*	*mphA*	*marA*	*bla*_TEM-220_	NF	NF	NF	NF	NF	NF	*bacA, tolC, evgA*	Yes	Yes	IncQ1, IncFIA, Col156, Col(BS512)
8	O75:H5	1193	AMP, FOX, SXT, CIP, NA	H41	*aac*(3)-Ib, *aadA17*	*mphA*	*marA*	*bla*_TEM-220_	NF	NF	NF	NF	NF	NF	*bacA, tolC, evgA*	Yes	Yes	IncFIA, IncQ1, Col(BS512), Col156
9	O6:H1	73	AMP, FOX, SXT, CIP, NA	H30	NF	NF	*marA*	NF	NF	NF	NF	NF	NF	NF	*mexB*	No	No	IncX1
10	O6:H1	73	AMP, CTX	H30	NF	NF	*marA*	NF	NF	NF	NF	NF	NF	NF	*acrB*	No	No	NF
11	O25:H4	131	AMP, CTX, CRO, CHL, SXT, CIP, NA	H30	*aac*(3)-Ib, *aadA5*	*mphA*	*marA*	*bla*_CTX__-M-27/99_	NF	NF	NF	*sul1, sul2*	*tet(A)*	NF	*acrB, gadW, pmrF*	Yes	Yes	IncFIA, IncFII, Col156
12	O25:H4	131	AMP, CTX, CRO, SXT, CIP, NA	H30	*aac*(3)-Ib, *aadA5*	*mphA*	*marA*	*bla*_CTX__-M-27/99_	NF	NF	NF	*sul1, sul2*	*tet(A)*	NF	*acrB, gadW, pmrF*	Yes	Yes	IncFIA, IncFII, Col156
13	O55:H7	335	AMP, SXT, NA	H30	*aac*(3)-Ib	NF	*marA*	*bla*_TEM-220_	NF	NF	NF	*sul1*	*tet(A)*	*dfrA7*	*evgA*, *cpxA*, *gadW*	Yes	Yes	IncQ1
14	O55:H7	335	AMP, SXT, NA	H30	*aac*(3)-Ib	NF	*marA*	*bla*_TEM-220_	NF	NF	NF	*sul1*	*tet(A)*	*dfrA7*	*evgA*, *cpxA*	Yes	Yes	IncQ1
15	O55:H7	335	AMP, CTX, SXT, NA	H30	*aac*(3)-Ib	NF	*marA*	*bla*_TEM-220_	NF	NF	NF	*sul1*	*tet(A)*	*dfrA7*	*evgA*, *cpxA*	Yes	Yes	IncQ1
16	O55:H7	335	AMP, SXT, NA	H30	*aac*(3)-Ib	NF	*marA*	*bla*_TEM-220_	NF	NF	NF	*sul1*	*tet(A)*	*dfrA7*	*evgA*, *cpxA*	Yes	Yes	IncQ1
20	O25:H4	131	AMP, CTX, FOX, SXT, CIP, NA	H30	*aac*(3)-Ib, *aadA5*	*mphA*	*marA*	*bla*_CTX__-M-27/99_	NF	NF	NF	*sul1, sul2*	*tet(A)*	*dfrA17*	*acrB, gadW*	Yes	Yes	IncFIA, IncFII, Col156
21	O75:H5	1193	AMP, FOX, SXT, CIP, NA	H41	*aac*(3)-Ib	*mphA*	*marA*	*bla*_TEM-220_	NF	NF	NF	NF	NF	*dfrA17*	*evgA, tolC, bacA*	Yes	Yes	IncQ1, IncFIA, Col156, Col(BS512)
23	O25:H4	131	AMP, CTX, CRO, CAZ, FEP, CHL, SXT, CIP, NA	H30	*aac*(3)-Ib, *aadA5*	*mphA*	*marA*	*bla*_CTX__-M-27/99_	NF	NF	NF	*sul1, sul2*	*tet(A)*	*dfrA17*	*acrB, gadW*	Yes	Yes	IncFIA, IncFII, Col156
24	O25:H4	131	AMP, CTX, CRO, SXT, CIP, NA	H30	*aac*(3)-Ib, *aadA5*	*mphA*	*marA*	*bla*_CTX__-M-27/99_	NF	NF	NF	*sul1, sul2*	*tet(A)*	*dfrA17*	*acrB, gadW*	Yes	Yes	IncFIA, IncFII, Col156
60	O*:H5	1193	ND	H41	*aac*(3)-Ib, *aadA5*	*mphA*	*marA*	*bla*_TEM-220_	NF	NF	NF	NF	NF	*dfrA17*	*acrS, bacA, tolC*	Yes	Yes	IncQ1, IncFIA, Col156, Col(BS512)
63	O25:H4	131	ND	H30	*aac*(3)-Ib, *aadA5*	*mphA*	*marA*	*bla*_CTX__-M-27/99_	NF	NF	NF	*sul1, sul2*	*tet(A)*	*dfrA17*	*gadW*	Yes	Yes	IncFII, Col156, IncFIA
64	O25:H4	131	ND	H30	*aac*(3)-Ib, *aadA5*	*mphA*	*marA*	*bla*_CTX__-M-27/99_	NF	NF	NF	*sul1, sul2*	*tet(A)*	*dfrA17*	*gadW*	Yes	Yes	IncFIA, IncFII, Col156
67	O25:H4	131	ND	H30	*aac*(3)-Ib, *aadA5*	*mphA*	*marA*	*bla*_CTX-M-15/88_, *bla*_OXA-1/140_	*catB3*	NF	NF	*sul1*	*tet(A)*	*dfrA17*	*acrS, gadW*	Yes	Yes	IncFIA
68	O89:H4	44	ND	H54	*aac*(3)-Ib, *aadA5*	*mphA*	*marA*	*bla*_CTX-M-15/88_, *bla*_OXA-1/140_	*catB3*	NF	NF	*sul1*	*tet(A)*	*dfrA17*	*acrS, gadW*	Yes	Yes	IncFIA, IncFII
71	O25:H4	131	ND	H30	*aadA5*	NF	*marA*	*bla*_CTX-M-15/88_, *bla*_OXA-1/140_	*catB3*	*aac*(3)-Ib-cr	QnrB2	*sul1*	*tet(A)*	*dfrA17*	*gadW*	Yes	Yes	IncFIA, IncFII, IncY
72	O25:H4	131	ND	H30	*aadA5*	NF	*marA*	*bla*_CTX-M-15/88_, *bla*_OXA-1/140_	*catB3*	*aac*(3)-Ib-cr	NF	*sul2*	NF	*dfrA14*	*gadW*	Yes	Yes	IncFIA, IncFII
73	O25:H4	131	ND	H30	*aac*(3)-Ib, *aadA5*	*mphA*	*marA*	*bla*_CTX-M-15/88_, *bla*_OXA-1/140_	*catB3*	NF	NF	*sul1*	*tet(A)*	*dfrA17*	*acrS, gadW*	Yes	Yes	IncFIA, IncFII
74	O*:H6	648	ND	H30	*aac*(3)-Ib, *aadA5*	*mphA*	*marA*	*bla*_CTX-M-15/88_, *bla*_OXA-1/140_	*catB3*	NF	NF	*sul1*	*tet(A)*	*dfrA17*	*acrS, gadW*	Yes	Yes	IncFIA, IncFII
87	O*:H4	131	ND	H30	*aac*(3)-Ib, *aadA5*	*mphA*	*marA*	*bla*_CTX__-M-27/99_	NF	NF	NF	*sul1*	*tet(A)*	*dfrA17*	*gadW*	Yes	Yes	IncFIA, IncFII
97	O25:H4	131	ND	H30	*aac*(3)-Ib, *aadA5*	*mphA*	*marA*	*bla*_CTX__-M-27/99_	NF	NF	NF	*sul1*	*tet(A)*	*dfrA17*	*gadW*	Yes	Yes	IncFIA, IncFII, Col156
102	O*:H7	335	ND	H30	*aac*(3)-Ib	NF	*marA*	*bla*_TEM-220_	NF	NF	NF	*sul1*	*tet(A)*	*dfrA7*	*cpXxA, evgA*	Yes	Yes	IncQ1
103	O25:H4	131	ND	H30	*aac*(3)-Ib, *aadA5*	*mphA*	*marA*	*bla*_CTX__-M-27/99_	NF	NF	NF	*sul2*	*tet(A)*	*dfrA17*	NF	Yes	Yes	IncFIA, IncFII, Col156
105	O55:H7	335	ND	H30	*aac*(3)-Ib	NF	*marA*	*bla*_TEM-220_	NF	NF	NF	*sul1*	*tet(A)*	*dfrA7*	*cpxA, evgA, acrB*	Yes	Yes	IncQ1
106	O*:H5	1193	ND	H41	*aac*(3)-Ib	*mphA*	*marA*	*bla*_TEM-220_	NF	NF	NF	NF	NF	*dfrA17*	*evgA, tolC, mphA*	Yes	Yes	IncQ1, IncFIA, Col156
BN1	O75:H5	1193	ND	H41	*aac*(3)-Ib	*mphA*	*marA*	*bla*_TEM-220_	NF	NF	NF	NF	NF	*dfrA17*	*bacA, tolC, evgA*	Yes	Yes	IncQ1, IncFIA
BN12	O*:H9	410	ND	H41	*aadA9*, *aac*(3)-Ib	*mphA*	*marA*	*bla*_TEM-220_	NF	NF	NF	NF	NF	*dfrA17*	*emrR*	Yes	Yes	IncQ1, IncFIA
BN19	O25:H4	131	ND	H30	*aadA5*, *aac*(3)-Ib, *aac*(3)-Iic/d/e	NF	*marA*	*bla*_TEM-220_, *bla*_CTX-M-15_, *bla*_OXA-1_, *bla*_OXA-140_	*catB3*	*aac(6′)*-Ib-cr	NF	*sul1, sul2*	NF	NF	*kdpE, gadW*	Yes	Yes	IncFIA, IncFII
BN2	O156:H7	NF	ND	H22	*aac*(3)-Ib	NF	*marA*	NF	NF	NF	NF	*sul1*	*tet(A)*	*dfrA17*	NF	No	Yes	Col
BN20	O89:H9	10	ND	H54	*aadA9*, *aac*(3)-Ib	NF	*marA*	*bla*_TEM-220_	NF	NF	NF	*sul1*	*tet(A)*	*dfrA1*	*mdtP, msbA, acrB, baeS/R, yojI*	Yes	Yes	IncQ1
BN25	O6:H11	48	ND	H41	*aadA5*, *aac*(3)-Ib	NF	*marA*	NF	NF	NF	NF	*sul2*	NF	*dfrA17*	*yojI, pmrF, emrR, bacA, acrS/B/E, msbA, evgA, kdpE, mdtP, eptA*	No	Yes	IncHI2A
BN26	O9:H19	162*	ND	H30	NF	NF	*marA*	NF	NF	NF	NF	NF	*tet(A)*	NF	*emrR, mdtA*	No	Yes	NF
BN27	O*:H2	165	ND	H54	*aac(6′)*-Ib7, *aac*(3)-Ib, *aadA9*	NF	*marA*	*bla*_TEM-220_	NF	NF	*qnrS1*	*sul3*	*tet(A)*	NF	*tolC, mdtO, msbA, acrB, baeS/R, acrD, gadX, evgA, pmrF*	Yes	Yes	NF
BN3	O18:H49	212	ND	H30	*aac(6′)*-Ib7, *aac*(3)-Ib, *aadA9*	NF	*marA*	NF	NF	NF	NF	*sul1*	*tet(A)*	*dfrA15*	*emrR*	No	Yes	NF
BN37	O8:H17	10	ND	H41	*aac*(3)-Ib, *aadA5*	NF	*marA*	*bla*_TEM-220_	NF	NF	NF	*sul2*	*tet(A)*	*dfrA15*	*yojI, pmrF, emrR, bacA, acrS/B/E, msbA, evgA, kdpE, mdtP, eptA*	Yes	Yes	IncFIA, IncFII
BN38	O89:H10	617	ND	H41	NF	NF	*marA*	*bla*_CTX-M-15/88_, *bla*_OXA-1/140_	*catB3*	*aac(6′)*-Ib-cr	NF	*sul1, sul2*	NF	NF	*yojI, pmrF, emrR, bacA, acrS/B/E, msbA, evgA, kdpE, mdtP, eptA*	Yes	Yes	IncFIA, IncFII
BN41	O171:H21	155	ND	H30	*aac(6′)*-Ib7, *aadA9*	NF	*marA*	*bla*_TEM-220_	NF	NF	NF	*sul2*	*tet(A)*	NF	*emrR*	Yes	Yes	IncHI1A, IncHI1B, IncFIA
BN42	O29:H8	448	ND	H30	*aadA13*, *aac*(3)-Ib	NF	*marA*	*bla*_CTX-M-5_	NF	NF	NF	*sul2*	NF	*dfrA14*	*emrR, mphA*	Yes	Yes	IncI, IncFII, IncFIA IncX4
BN43	O*:H4	6161	ND	H30	NF	NF	*marA*	NF	NF	NF	NF	*sul2*	NF	*dfrA14*	*baeS*	No	Yes	NF
BN44	O8:H9	410	ND	H41	*aac(6′)*-Ib7, *aadA9*	*mphA*	*marA*	*bla*_CTX-M-5_, *bla*_OXA-1_, *bla*_TEM-30/220_	*catB3*	*aac(6′)*-Ib-cr	NF	NF	NF	NF	*emrR*	Yes	Yes	IncFIA, IncQ1
BN47	O89:H9	10	ND	H54	*acrD*/E/F, *aadA9*, *aac*(3)-Ib, *aac(6′)*-Ib7	NF	*marA*	*bla*_TEM-220_	NF	NF	NF	*sul1*	NF	*dfrA1*	*gadX, tolC, mdtF/N/O, emrK*	Yes	Yes	Col440II, IncQ1
BN48	O17:H11	NF	ND	H41	*aac*(3)-Ib	NF	*marA*	*bla*_TEM-7/75/177_	NF	NF	NF	*sul1*	NF	*dfrA7*	*axyY*	Yes	Yes	IncQ1
BN49	O45:H11	10	ND	H41	NF	NF	*marA*	NF	NF	NF	*qnrB19*	NF	NF	NF	*yojI, pmrF, emrR, bacA, acrS/B/E, msbA, evgA, kdpE, mdtP, eptA*	No	No	Col
BN50	O*:H4	167	ND	H30	NF	NF	*marA*	NF	NF	NF	NF	*sul2*	NF	*dfrA14*	*baeS*	No	Yes	NF
BN51	O171:H21	6161	ND	H30	*aac(6′)*-Ib7, *aadA9*	NF	*marA*	*bla*_TEM-220_	NF	NF	NF	*sul2*	*tet(A)*	NF	*mdtA/B, emrR*	Yes	Yes	IncHI1A, IncHI1B, IncFIA
BN55	O185:H8	155	ND	H30	*acrB*, *aadA1*, *aac*(3)-Ib, *aac(6′)*-Ib7	NF	*marA*	*bla*_TEM-220_	NF	NF	NF	*sul1*	*tet(A)*	*dfrA1*	*yojI*, *pmrF*, *emrR*, *bacA*, *acrS/B/E*, *msbA*, *evgA*, *kdpE*, *mdtP*, *eptA*, *emtK*, *cpxA*	Yes	Yes	IncFIB, IncQ1
BN56	O*:H7	2163	ND	H30	NF	NF	*marA*	NF	NF	NF	NF	NF	NF	NF	*emrR, baeS*	No	No	NF
BN57	O9:H19	162*	ND	H30	NF	NF	*marA*	NF	NF	NF	NF	*sul2*	*tet(A)*	NF	*mdtA, emrR*	No	Yes	NF
BN6	O6:H1	73	ND	H22	*aac*(3)-Ib	NF	*marA*	*bla*_TEM-220_	NF	NF	NF	*sul1*	*tet(A)*	*dfrA7*	NF	Yes	Yes	IncFIB

**Table 3:** Genomic characteristics of uropathogenic *K. pneumoniae* strains isolated in this study for acquired AMR-associated genes and plasmid replicon types. NF means not found; ‘Yes’ means the strain is either ESBL-producing or MDR; and ‘No’ means the sample is either non-ESBL or non-MDR.

Sample Name	Sequence Type Pasteur	AMR Phenotype	Aminoglycosides	Ciplrofloxacin	Penicillins + β-lactamase Inhibitors	ESBL Cephalosporins	Phenicols	Fluoroquinolones	Quinolone	Sulfonamide	Tetracycline	Folate Pathway Inhibitors	Antibiotic Effulx/Regulation	ESBL	MDR	Plasmid Replicon
25	11	ND	*aac(6′)*-30/*aac(6′)*-Ib’/7/10, *aadA9*	NF	*bla*_LEN-4/6_	*bla*_SHV-28_, *bla*_CTX-M-15_, *bla*_OXA-1/140_, *bla*_NDM-1_	*catB3*	*oqxB*, *aac(6′)*-Ib-cr	*qnrB9*	*sul2*	NF	NF	NF	Yes	Yes	IncFII, IncFIB, IncR
26	11	ND	*aac(6′)*-30/*aac(6′)*-Ib’/7/10, *aadA9*	NF	*bla*_LEN-4/6_	*bla*_SHV-28_, *bla*_CTX-M-15_, *bla*_OXA-1/140_, *bla*_NDM-1_	*catB3*	*oqxB*, *aac(6′)*-Ib-cr	*qnrB9*	*sul2*	NF	NF	NF	Yes	Yes	IncFII, IncFIB, IncR
27	11	ND	*aac(6′)*-30/*aac(6′)*-Ib’/7/10, *aadA9*	NF	*bla*_LEN-4/6_	*bla*_SHV-28_, *bla*_CTX-M-15_, *bla*_OXA-1/140_, *bla*_NDM-1_	*catB3*	*oqxB*, *aac(6′)*-Ib-cr	*qnrB9*	*sul2*	NF	NF	NF	Yes	Yes	IncFII, IncFIB, IncR
28	11	ND	*aac(6′)*-30/*aac(6′)*-Ib’/7/10, *aadA9*	NF	*bla*_LEN-4/6_	*bla*_SHV-28_, *bla*_CTX-M-15_, *bla*_OXA-1/140_, *bla*_NDM-1_	*catB3*	*oqxB*, *aac(6′)*-Ib-cr	*qnrB9*	*sul2*	NF	NF	NF	Yes	Yes	IncFII, IncFIB, IncR
29	11	ND	*aac(6′)*-30/*aac(6′)*-Ib’/7/10, *aadA9*	NF	*bla*_LEN-4/6_	*bla*_SHV-28_, *bla*_CTX-M-15_, *bla*_OXA-1/140_, *bla*_NDM-1_	*catB3*	*oqxB*, *aac(6′)*-Ib-cr	*qnrB9*	*sul2*	NF	NF	NF	Yes	Yes	IncFII, IncFIB, IncR
30	11	ND	*aac(6′)*-30/*aac(6′)*-Ib’/7/10, *aadA9*	NF	*bla*_LEN-4/6_	*bla*_SHV-28_, *bla*_CTX-M-15_, *bla*_OXA-1/140_, *bla*_NDM-1_	*catB3*	*oqxB*, *aac(6′)*-Ib-cr	*qnrB9*	*sul2*	NF	NF	NF	Yes	Yes	IncFII, IncFIB, IncR
31	11	ND	*aac(6′)*-30/*aac(6′)*-Ib’/7/10, *aadA9*	NF	*bla*_LEN-4/6_	*bla*_SHV-28_, *bla*_CTX-M-15_, *bla*_OXA-1/140_, *bla*_NDM-1_	*catB3*	*oqxB*, *aac(6′)*-Ib-cr	*qnrB9*	*sul2*	NF	NF	NF	Yes	Yes	IncFII, IncFIB, IncR
32	11	ND	*aac(6′)*-30/*aac(6′)*-Ib’/7/10, *aadA9*	NF	*bla*_LEN-4/6_	*bla*_SHV-28_, *bla*_CTX-M-15_, *bla*_OXA-1/140_, *bla*_NDM-1_	*catB3*	*oqxB*, aac(6′)-Ib-cr	*qnrB9*	*sul2*	NF	NF	NF	Yes	Yes	IncFII, IncFIB, IncR
33	11	ND	*aac(6′)*-30/*aac(6′)*-Ib’/7/10, *aadA9*	NF	*bla*_LEN-4/6_	*bla*_SHV-28_, *bla*_CTX-M-15_, *bla*_OXA-1/140_, *bla*_NDM-1_	*catB3*	*oqxB*, aac(6′)-Ib-cr	*qnrB9*	*sul2*	NF	NF	NF	Yes	Yes	IncFII, IncFIB, IncR
34	11	ND	*aac(6′)*-30/*aac(6′)*-Ib’/7/10, *aadA9*	NF	*bla*_LEN-4/6_	*bla*_SHV-28_, *bla*_CTX-M-15_, *bla*_OXA-1/140_, *bla*_NDM-1_	*catB3*	*oqxB*, *aac(6′)*-Ib-cr	*qnrB9*	*sul2*	NF	NF	NF	Yes	Yes	IncFII, IncFIB, IncR
35	11	ND	*aac(6′)*-30/*aac(6′)*-Ib’/7/10, *aadA9*	NF	*bla*_LEN-4/6_	*bla*_SHV-28_, *bla*_CTX-M-15_, *bla*_OXA-1/140_, *bla*_NDM-1_	*catB3*	*oqxB*, *aac(6′)*-Ib-cr	*qnrB9*	*sul2*	NF	NF	NF	Yes	Yes	IncFII, IncFIB, IncR
36	11	ND	*aac(6′)*-30/*aac(6′)*-Ib’/7/10, *aadA9*	NF	*bla*_LEN-4/6_	*bla*_SHV-28_, *bla*_CTX-M-15_, *bla*_OXA-1/140_, *bla*_NDM-1_	*catB3*	*oqxB*, *aac(6′)*-Ib-cr	*qnrB9*	*sul2*	NF	NF	NF	Yes	Yes	IncFII, IncFIB, IncR
55	11	ND	*aac(6′)*-30/*aac(6′)*-Ib’/7/10, *aadA9*	NF	*bla*_LEN-4/6_	*bla*_SHV-28_, *bla*_CTX-M-15_, *bla*_OXA-1/140_, *bla*_NDM-1_	*catB3*	*oqxB*, *aac(6′)*-Ib-cr	*qnrB9*	*sul2*	NF	NF	NF	Yes	Yes	IncFII, IncFIB, IncR
56	11	ND	*aac(6′)*-30/*aac(6′)*-Ib’/7/10, *aadA9*	NF	*bla*_LEN-4/6_	*bla*_SHV-28_, *bla*_CTX-M-15_, *bla*_OXA-1/140_, *bla*_NDM-1_	*catB3*	*oqxB*, *aac(6′)*-Ib-cr	*qnrB9*	*sul2*	NF	NF	NF	Yes	Yes	IncFII, IncFIB, IncR
89	11	ND	*aac(6′)*-30/*aac(6′)*-Ib’/7/10, *aadA9*	NF	*bla*_LEN-4/6_	*bla*_SHV-28_, *bla*_CTX-M-15_, *bla*_OXA-1/140_, *bla*_NDM-1_	*catB3*	*oqxB*, *aac(6′)*-Ib-cr	*qnrB9*	*sul2*	NF	NF	NF	Yes	Yes	IncFII, IncFIB, IncR
90	NF	ND	*aac(6′)*-Ib7, *aadA9*	NF	*bla*_LEN-3/4/5/6_	*bla*_SHV-28_, *bla*_CTX-M-15_, *bla*_OXA-1/140_	NF	NF	NF	*sul1*	*tet*(A)	*dfrA17*	*yojI, pmrF, emrR, bacA, acrB, msbA, evgA, kdpE, mdtP, eptA, emtK, cpxA*	Yes	Yes	IncN
BN14	0b8e	ND	*aac(6′)*-30/*aac(6′)*-Ib’/7/10, *aadA9*	*marA*	*bla*_LEN-3/4/5/6_	*bla*_SHV-28_, *bla*_CTX-M-15_, *bla*_TEM-220_	*catB3*	*oqxA*/B, *aac(6′)*-Ib-cr	*qnrB6*/17	*sul1*	*tet*(B)	*dfrA27*	*yojI, pmrF, emrR, bacA, acrB, msbA, evgA, kdpE, mdtP, eptA, emtK, cpxA*	Yes	Yes	IncR, IncFII, IncFIA, Col, IncX4
BN16	67b2	ND	*aac(6′)*-30/*aac(6′)*-Ib’/7/10, *aadA9*	*marA*	*bla*_LEN-3/4/5/6_	*bla*_CTX-M-15/88_	*catB3*	*oqxA*/B, *aac(6′)*-Ib-cr	*qnrB6*/17	*sul1, sul2*	*tet*(B)	*dfrA27*	*yojI, pmrF, emrR, bacA, acrB, msbA, evgA, kdpE, mdtP, eptA, emtK, cpxA*	Yes	Yes	IncR, IncFII, IncFIA, Col, IncX4
BN7	6b6f	ND	*aac(6′)*-30/*aac(6′)*-Ib’/7/10, *aadA9*	NF	NF	NF	NF	*oqxA*/B, *aac(6′)*-Ib-cr	*qnrB6*/17	NF	NF	NF	NF	No	Yes	IncR

## Data Availability

Raw and generated data for this Pilot study are available in the following links: https://doi.org/10.6084/m9.figshare.17105876 (supplementary files, accessed on 16 November 2021), https://doi.org/10.6084/m9.figshare.17091293 (draft genome assemblies, accessed on 16 November 2021) and https://doi.org/10.6084/m9.figshare.11965455 (Table 2 and Table 3, accessed on16 November 2021).

## Supplementary Materials

The following are available online at https://www.mdpi.com/article/10.3390/antibiotics10121547/s1. Table S1: Metadata of E. coli and K. pneumoniae strains isolated from urine samples including Antibiotic Sensitivity Test results of *n* = 16 isolates; Table S2: Comparison of host niche, disease implication, isolation source, collection year, antimicrobial and virulence gene contents between three most abundant E. coli clones, ST131, ST335 and ST10 (a) and K. pneumoniae ST11 (b) strains in our study and selected isolate genomes listed in BacWGSTdb 2.0. ND means not determined; Figure S1: Distribution of antimicrobial resistance genes (AMRGs; right panel) among E. coli (a) and K. pneumoniae (b) isolates from our HATUA Pilot collection. Left panel shows clustering of the strains in a phylogenetic tree according to the presence (green blocks) or absence (pink blocks) of AMRGs; Figure S2: Pairwise SNP distances in core genome multi-locus sequence type (cgMLST)-based alleles of the three most abundant E. coli clones, ST131, ST335, and ST10 (a) and K. pneumoniae ST11 (b) strains in our study and selected isolate genomes listed in BacWGSTdb. The assemblies of the reference sequences were downloaded from European Nucleotide Archive.
