# Supplementary material for: Pan-Resistome Characterization of Uropathogenic Escherichia coli and Klebsiella pneumoniae Strains Circulating in Uganda and Kenya, Isolated from 2017–2018
Source: Antibiotics (Basel). 2021 Dec 17;10(12):1547. doi: 10.3390/antibiotics10121547 (PMC8698711; doi:10.3390/antibiotics10121547)
Supplement: Supplementary file 1 [file antibiotics-10-01547-s001.zip › Supplementary_Figure_2a.pdf]

(a)

ST131

	103	11	12	20	23	24	5	63	64	67	6	71	72	73	87	97	BN19	CP023853.1	JNQU01	NIDR01	SDUG01	SGIW01
103	0	223	255	243	289	314	275	271	286	1326	312	1680	1281	743	265	200	1342	1363	6774	11578	11616	1799
11	223	0	221	225	267	280	209	268	269	1314	290	1670	1265	746	236	188	1320	1350	6767	11566	11594	1763
12	255	221	0	226	282	307	265	273	258	1318	293	1659	1270	745	225	197	1334	1371	6766	11574	11601	1784
20	243	225	226	0	254	273	216	247	262	1313	260	1661	1238	731	213	175	1320	1348	6771	11573	11616	1775
23	289	267	282	254	0	301	286	301	300	1373	309	1721	1292	765	283	239	1374	1391	6812	11614	11647	1820
24	314	280	307	273	301	0	295	308	317	1382	338	1698	1307	796	312	264	1389	1412	6839	11629	11680	1847
5	275	209	265	216	286	295	0	276	297	1343	304	1701	1272	744	266	213	1342	1370	6792	11582	11630	1799
63	271	268	273	247	301	308	276	0	263	1321	321	1698	1269	744	246	224	1353	1394	6801	11605	11643	1817
64	286	269	258	262	300	317	297	263	0	1358	303	1721	1282	777	259	237	1366	1391	6800	11599	11645	1822
67	1326	1314	1318	1313	1373	1382	1343	1321	1358	0	1371	1355	1345	1344	1291	1276	1475	910	6975	11310	11140	1335
6	312	290	293	260	309	338	304	321	303	1371	0	1744	1315	808	279	246	1367	1399	6784	11640	11667	1838
71	1680	1670	1659	1661	1721	1698	1701	1698	1721	1355	1744	0	1525	1513	1668	1652	1657	1145	7075	11359	10647	1678
72	1281	1265	1270	1238	1292	1307	1272	1269	1282	1345	1315	1525	0	995	1227	1229	1122	1350	6646	11549	11475	1819
73	743	746	745	731	765	796	744	744	777	1344	808	1513	995	0	732	704	1150	1386	6624	11584	11523	1857
87	265	236	225	213	283	312	266	246	259	1291	279	1668	1227	732	0	207	1317	1340	6741	11581	11614	1775
97	200	188	197	175	239	264	213	224	237	1276	246	1652	1229	704	207	0	1275	1313	6731	11541	11580	1752
BN19	1342	1320	1334	1320	1374	1389	1342	1353	1366	1475	1367	1657	1122	1150	1317	1275	0	1489	6811	11634	11587	1928
CP023853.1	1363	1350	1371	1348	1391	1412	1370	1394	1391	910	1399	1145	1350	1386	1340	1313	1489	0	6764	11145	10996	1092
JNQU01	6774	6767	6766	6771	6812	6839	6792	6801	6800	6975	6784	7075	6646	6624	6741	6731	6811	6764	0	12594	12716	7017
NIDR01	11578	11566	11574	11573	11614	11629	11582	11605	11599	11310	11640	11359	11549	11584	11581	11541	11634	11145	12594	0	4557	11380
SDUG01	11616	11594	11601	11616	11647	11680	11630	11643	11645	11140	11667	10647	11475	11523	11614	11580	11587	10996	12716	4557	0	11114
SGIW01	1799	1763	1784	1775	1820	1847	1799	1817	1822	1335	1838	1678	1819	1857	1775	1752	1928	1092	7017	11380	11114	0

ST335

	102	105	13	14	15	16	AOTA01	MINC01	NKDG01	SGUH01	WJRH01
102	0	718	733	821	769	783	5117	5629	5906	5317	5360
105	718	0	153	262	242	254	4651	5145	5450	4881	4888
13	733	153	0	265	271	222	4654	5134	5433	4870	4879
14	821	262	265	0	214	187	4712	5210	5513	4944	4951
15	769	242	271	214	0	192	4728	5222	5487	4942	4953
16	783	254	222	187	192	0	4700	5204	5507	4932	4941
AOTA01	5117	4651	4654	4712	4728	4700	0	1673	2587	1412	1457
MINC01	5629	5145	5134	5210	5222	5204	1673	0	2870	1026	867
NKDG01	5906	5450	5433	5513	5487	5507	2587	2870	0	2816	2843
SGUH01	5317	4881	4870	4944	4942	4932	1412	1026	2816	0	957
WJRH01	5360	4888	4879	4951	4953	4941	1457	867	2843	957	0

ST10

	BIBD01	BN20	BN37	BN47	BN49	CXYF01	JHDI01	JMGV01	LSBS01	MOZS01
BIBD01	0	7737	6036	7672	5443	6021	8225	6275	7646	9865
BN20	7737	0	7250	251	9778	7340	8773	8175	2070	8867
BN37	6036	7250	0	7251	8381	6934	9269	7805	7439	9909
BN47	7672	251	7251	0	9736	7267	8738	8103	2009	8844
BN49	5443	9778	8381	9736	0	8488	10458	8651	9667	12072
CXYF01	6021	7340	6934	7267	8488	0	8033	7143	7381	9575
JHDI01	8225	8773	9269	8738	10458	8033	0	8587	8745	10922
JMGV01	6275	8175	7805	8103	8651	7143	8587	0	7897	10069
LSBS01	7646	2070	7439	2009	9667	7381	8745	7897	0	8765
MOZS01	9865	8867	9909	8844	12072	9575	10922	10069	8765	0
