# Supplementary material for: Pan-Resistome Characterization of Uropathogenic Escherichia coli and Klebsiella pneumoniae Strains Circulating in Uganda and Kenya, Isolated from 2017–2018
Source: Antibiotics (Basel). 2021 Dec 17;10(12):1547. doi: 10.3390/antibiotics10121547 (PMC8698711; doi:10.3390/antibiotics10121547)
Supplement: Supplementary file 1 [file antibiotics-10-01547-s001.zip › Supplementary_Figure_2b.pdf]

(b)

25	26	27	28	29	30	31	32	33	34	35	36	55	56	89	90	BN14	BN16	BN7	LXMM01	MZZV01	NBOT01	VONS01	VUBS01	
0	251	4654	269	305	327	317	291	302	288	322	310	307	237	389	126342	4711	4711	25328	5043	5695	5440	4989	4888	25
251	0	4621	248	257	296	274	290	299	283	309	325	305	206	356	126322	4679	4679	25304	5019	5635	5396	4949	4873	26
4654	4621	0	4625	4665	4685	4679	4647	4619	4615	4652	4622	4635	4579	4708	124320	62	62	24519	3575	4346	3998	3528	3311	27
269	248	4625	0	271	290	267	236	272	260	284	291	275	202	349	126311	4683	4683	25302	4997	5645	5400	4943	4848	28
305	257	4665	271	0	325	324	337	336	311	350	361	342	252	410	126362	4723	4723	25354	5045	5687	5448	4993	4917	29
327	296	4685	290	325	0	293	320	325	265	306	319	287	260	384	126366	4743	4743	25371	5071	5679	5440	5013	4934	30
317	274	4679	267	324	293	0	321	300	259	269	281	255	238	350	126371	4737	4737	25354	5053	5655	5432	4995	4924	31
291	290	4647	236	337	320	321	0	312	294	325	325	319	250	397	126338	4705	4705	25317	5041	5691	5434	4977	4890	32
302	299	4619	272	336	325	300	312	0	248	285	228	258	230	345	126310	4677	4677	25304	5019	5650	5385	4965	4878	33
288	283	4615	260	311	265	259	294	248	0	214	230	195	217	313	126308	4673	4673	25309	4997	5597	5370	4943	4862	34
322	309	4652	284	350	306	269	325	285	214	0	225	199	271	304	126340	4710	4710	25340	5030	5629	5404	4962	4865	35
310	325	4622	291	361	319	281	325	228	230	225	0	236	235	307	126320	4680	4680	25314	5020	5620	5369	4952	4871	36
307	305	4635	275	342	287	255	319	258	195	199	236	0	235	297	126331	4693	4693	25327	5029	5627	5392	4971	4882	55
237	206	4579	202	252	260	238	250	230	217	271	235	235	0	326	126281	4637	4637	25276	4975	5603	5352	4907	4846	56
389	356	4708	349	410	384	350	397	345	313	304	307	297	326	0	126387	4766	4766	25367	5110	5722	5477	5046	4962	89
126342	126322	124320	126311	126362	126366	126371	126338	126310	126308	126340	126320	126331	126281	126387	0	124289	124289	133975	126081	126619	126387	125858	125762	90
4711	4679	62	4683	4723	4743	4737	4705	4677	4673	4710	4680	4693	4637	4766	124289	0	0	24519	3519	4290	3942	3472	3255	BN14
4711	4679	62	4683	4723	4743	4737	4705	4677	4673	4710	4680	4693	4637	4766	124289	0	0	24519	3519	4290	3942	3472	3255	BN16
25328	25304	24519	25302	25354	25371	25354	25317	25304	25309	25340	25314	25327	25276	25367	133975	24519	24519	0	25581	25914	25488	25345	25254	BN7
5043	5019	3575	4997	5045	5071	5053	5041	5019	4997	5030	5020	5029	4975	5110	126081	3519	3519	25581	0	4378	4301	3411	3329	LXMM01
5695	5635	4346	5645	5687	5679	5655	5691	5650	5597	5629	5620	5627	5603	5722	126619	4290	4290	25914	4378	0	4000	3732	4122	MZZV01
5440	5396	3998	5400	5448	5440	5432	5434	5385	5370	5404	5369	5392	5352	5477	126387	3942	3942	25488	4301	4000	0	4113	4115	NBOT01
4989	4949	3528	4943	4993	5013	4995	4977	4965	4943	4962	4952	4971	4907	5046	125858	3472	3472	25345	3411	3732	4113	0	515	VONS01
4888	4873	3311	4848	4917	4934	4924	4890	4878	4862	4865	4871	4882	4846	4962	125762	3255	3255	25254	3329	4122	4115	515	0	VUBS01

ST11
