# Supplementary figures and images for: Pan-Resistome Characterization of Uropathogenic Escherichia coli and Klebsiella pneumoniae Strains Circulating in Uganda and Kenya, Isolated from 2017–2018
Source: Antibiotics (Basel). 2021 Dec 17;10(12):1547. doi: 10.3390/antibiotics10121547 (PMC8698711; doi:10.3390/antibiotics10121547)

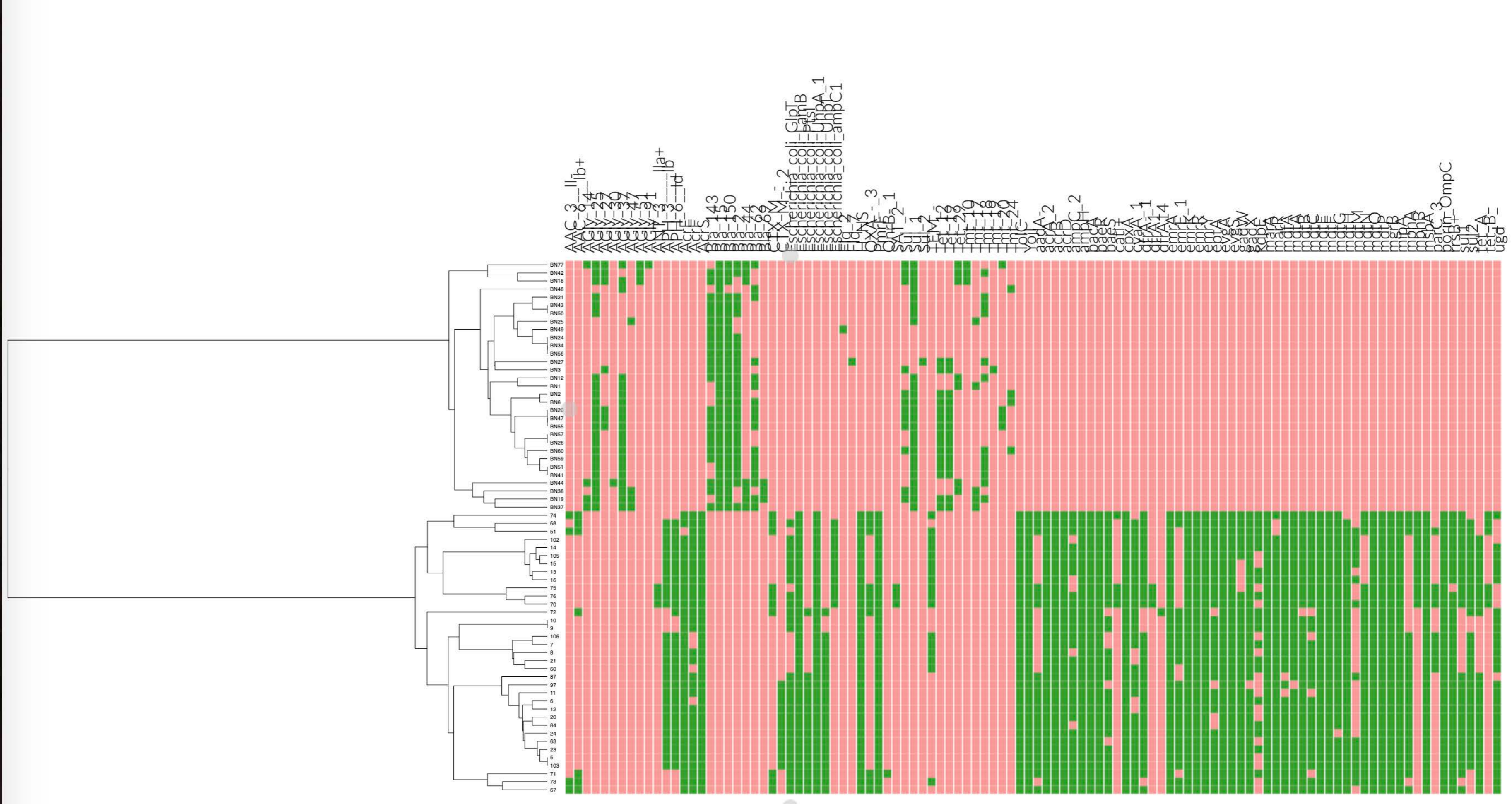

Supplement: Supplementary file 1 [file antibiotics-10-01547-s001.zip › Supplementary_Figure_1a.pdf]

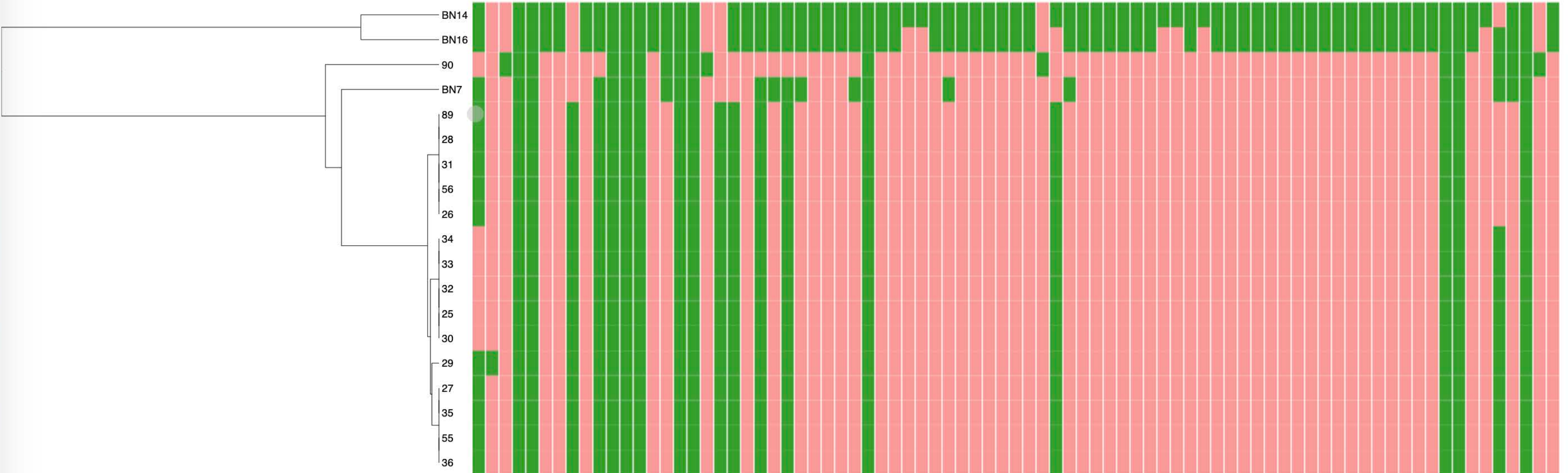

Supplement: Supplementary file 1 [file antibiotics-10-01547-s001.zip › Supplementary_Figure_1b.pdf]
